# Six-month intervention effect of a digital movement behavior intervention on parent- and child intermediary outcomes—results from *the Let’s Grow* randomized controlled trial

**DOI:** 10.1186/s12966-025-01764-1

**Published:** 2025-06-16

**Authors:** Johanna Sandborg, Katherine L. Downing, Liliana Orellana, Rachael W. Taylor, Lisa M. Barnett, Valerie Carson, Kylie D. Hesketh

**Affiliations:** 1 Institute for Physical Activity and Nutrition, Deakin University, Faculty of Health, Geelong, VIC Australia; 2 Biostatistics Unit, Faculty of Health, Deakin University, Geelong, VIC Australia; 3 Department of Medicine, University of Otago, Dunedin, New Zealand; 4 School of Health and Social Development, Deakin University, Geelong, VIC Australia; 5 Faculty of Kinesiology, Sport, and Recreation, University of Alberta, Edmonton, AB Canada

**Keywords:** MHealth, Parenting practices, Movement behaviors, Self-efficacy, Confidence, Knowledge, Mediators, Motor skills, Emotional regulation

## Abstract

**Background:**

Parental-focused interventions often aim to improve child health behaviors by changing parenting practices and cognitions and supporting child skill development. These intermediary outcomes serve as milestones that indicate progress towards achieving the ultimate intervention goal; however, the impact on these is rarely reported. The aim of this study was to investigate the effect of a digital intervention, intended to help parents promote healthy movement behaviors in toddlers on these intermediary outcomes.

**Methods:**

This study utilized data from the *Let’s Grow* trial (*n* = 1165). Participants were recruited Australia-wide and randomized to usual care (routine child healthcare visits) or intervention (usual care plus *Let’s Grow* app) following baseline assessment. Participants with data on at least one intermediary outcome (assessed via an online survey) at baseline and mid-intervention (6-months) were included (usual care, *n* = 618; intervention, *n* = 547). These included parental cognitions (knowledge, self-efficacy, confidence) and behaviors (co-participation, role modelling, family rules and routines, screens in child’s bedroom), and child developmental skills (motor skills, emotional regulation). Linear regression compared between-group outcomes. We also explored whether changes in the intermediary outcomes were associated with intervention engagement (Web app analytics).

**Results:**

The intervention group had higher knowledge of child movement behaviors (mean difference = 0.41, *P* = 0.002) compared to control. This difference was driven by knowledge in physical activity (mean differences 0.12, *P* = 0.028) and sleep (mean difference 0.27, *P* = 0.003) topics. No significant effect was observed for the other intermediary outcomes. Higher engagement was associated with improvements in parental knowledge of child movement behaviors and physical activity, confidence, ease of parenting, family rules for movement behaviors and screen time, and less parental screen time (all *P* ≤ 0.039).

**Conclusions:**

While *Let’s Grow* positively influenced physical activity and sleep knowledge at the mid-intervention point, our findings suggests that parents might need more time or support to improve cognitions and behaviors related to children’s sedentary behavior/screen time and child developmental skills. Further clarity on whether the observed changes translate into differential impacts on child movement behaviors will be reported following trial conclusion. Engagement appears to enhance intervention effects, highlighting the importance of strategies to optimize engagement.

**Trial registration:**

ACTRN12620001280998; U1111-1252–0599.

## Background

Early childhood (0–5 years) is an important time to promote healthy behaviors [1]. In the early years parents play a key role in providing opportunities, encouragement, and support to positively influence children’s movement behaviors (i.e., physical activity, sedentary behavior and sleep) [2, 3]. Moreover, parental practices (e.g., parental modeling of behavior, rules and routines) and cognitions (e.g., self-efficacy, knowledge, confidence) have been found to be important contributors to physical activity and sedentary behavior [2, 4–7], including screen use [2, 8], and sleep [5] in early childhood. Child developmental skills, including motor skill competence [9] and emotional self-regulation [10], are also important for development of healthy behaviors. Motor skills are linked to better physical activity levels [11], while emotional self-regulation supports mental health, healthy living and learning ability [12, 13]. Therefore, targeting these skills in early childhood can significantly enhance overall development and well-being. These have all also been identified as important intermediary outcomes in early childhood obesity prevention interventions [14], representing milestones that indicate progress towards achieving the desired primary outcomes e.g., positive change in child health behaviors.

Examining intervention effects on these intermediary outcomes, which are important for promoting child movement behaviors, can help us to understand how intervention effects are achieved and can further inform the development of more impactful intervention strategies. Although early childhood interventions are often designed to change intermediary outcomes such as parental behaviors that would in turn influence their children’s outcomes [15, 16], reporting of the intervention effect on these intermediary outcomes is limited. Moreover, previous studies have mainly focused only on a single movement behavior [17–25], nutrition [22, 23] or motor skills [26], or targeted preschool- [19, 20, 25, 26] and school-aged children [17, 18, 21–24]. Considering the co-dependency of movement behaviors [27], where more time spent in one behavior reduces time available for others, targeting all behaviors presents an enhanced opportunity for behavior change and is potentially more effective for promoting healthy development in young children. To date, no previous study has reported the effects of an intervention targeting all movement behaviors on parental cognitions and behaviors and child developmental skills in younger children (< 3 years).

The *Let’s Grow* trial is a 12-month stand-alone parenting mHealth intervention delivered via learning modules that parents work through over time, designed to improve the composition of movement behaviors in 2-year-old children [28]. More specifically, the purpose of the intervention was to change the hypothesized intermediary outcomes to improve movement behaviors in children, i.e., parental cognitions (knowledge, self-efficacy, and confidence) and behaviors (modeling of behaviors, rules, and routines), as well as support child developmental skills (motor skills and emotional regulation). This paper aims to investigate the effect of the *Let’s Grow* intervention on these intermediary outcomes after 6 months (i.e., mid-intervention) to determine whether the intervention is working in the way intended and to inform the development of more impactful interventions. In addition, we aimed to investigate whether any change in these intermediary outcomes between baseline and mid-intervention was associated with levels of engagement (defined as total time spent in the *Let’s Grow* app and number of completed learning modules) among participants in the intervention group.

## Methods

### Study design

This study uses baseline and 6-month data from the *Let’s Grow* randomized controlled trial conducted between March 2021 and June 2024. The full study design and rationale have been described in detail previously [28, 29]. In short, the trial investigated the effectiveness of a 12-month digital intervention (the *Let’s Grow* app) intended to support parents in improving movement behaviors in their 2-year-olds. Assessments occurred at baseline, 6-months (mid-intervention), 12-months (completion of the intervention) and 24-months post baseline (1 year post intervention) via an online survey (REDCap), as well as collection of 24-h movement behavior data via accelerometry. This manuscript focuses on the intermediary outcomes including parent cognitions and behaviors and child developmental skills (motor skills and emotional regulation) at baseline and 6-months. The trial was registered in advance with the Australian New Zealand Clinical Trials Registry (trial registration nr; ACTRN12620001280998; U1111-1252–0599) and ethical approval was obtained by the Deakin University Human Research Ethics Committee, Australia (2020–077). The study is reported according to the Consolidated Standards of Reporting Trials of Electronic and Mobile Health Applications and online Telehealth (CONSORT-EHEALTH) statement [30].

### Participants

Families were recruited through social media (e.g., Facebook, parenting blogs) between March 2021 and June 2022. Parents were eligible if they were 18 years or older, resided in Australia, owned a mobile phone, had internet access, were literate in English and had a child aged 18–35 months who was walking independently and was not diagnosed with or receiving treatment for a sleep disorder. Eligible parents received details of the study, including that the study aimed to test an online program for parents of 2-year-olds, focusing on skills to support healthy child development (managing play, screen time and sleep), and provided written informed consent.

### Randomization and blinding

After completion of the baseline survey, participants were randomized (1:1 ratio) stratified by geographical location (urban or outer/remote for each of the eight Australian states/territories; 16 strata) to either the control or intervention group. The random allocation sequences were computer generated and embedded in REDCap (Vanderbilt, USA) to ensure allocation concealment. Participants were not blinded to the allocation due to the nature of the intervention. All participants (intervention and control) received a $20 retail gift vouch upon completion of each assessment point.

### Study treatments

The control group received usual care (e.g., visits with maternal, child and family health nurses, general practitioner visits; varies by state/territory of residence) as well as eight electronic bulletins (‘Toddler Tips’) via email on unrelated topics (e.g., basic child first aid, language development, toilet training) every six weeks during the intervention period. In addition to usual care, parents allocated to the intervention group received access to the *Let’s Grow* app (a mobile web app with linked SMS notifications). The app was developed by the School of IT Deakin University and, apart from fixing back-end bugs that caused outages, the same version of the app was used for the duration of the trial. Participants allocated to the intervention received information on how to download and log into the app. The aim of the intervention was to change the hypothesized intermediary outcomes to ultimately improve children’s movement behaviors. The intervention content was designed using Michie’s Behavior Change Wheel [31], employing behavior change techniques e.g., goal setting, self-monitoring [32], designed to target the parent intermediary outcomes of interest [33, 34] and including practical advice, tips and tools (e.g., information, government guidelines, parenting strategies) to help parents improve their child’s movement behaviors. The app was built around eight learning modules (i.e., information and tips in text and video format on child sleep, play, screen time and general parenting), included several functions (i.e., a toolkit with information and parenting strategies, and a community chat forum), and participants received text messages linked to the app. The toolkit and community chat forum were freely accessible throughout the intervention period. Parents were free to choose which of the eight modules they wished to work on and could only work on one module at a time. Modules covered parenting and behavior change strategies for multiple target child behaviors and focused on the interplay between these (e.g., increasing physical activity to decrease sedentary time). Each module included a set of activities with linked SMS notifications to assist completion of activities (e.g., goal monitoring) and had a minimum time frame (ranging from 2–5 weeks) to ensure enough time to complete the behavior change activities taught within the module before moving on the next module. A module was considered complete when the app verified that all components were completed including watching the video(s), seeing the information (as confirmed by clicking on each page) and completing each of the activities, and the parent clicked a button indicating they had completed the module. After completing one module, participants could choose a new module to work on and the completed modules remained accessible. The minimum timeframe to complete all modules was 22 weeks; however, participants were not expected to complete the program in this time as the intervention duration was 12 months.

### Participant demographics

Participants provided family demographics at baseline via an online survey. Main carer characteristics included age, relationship to the child (mother/father), birth country (born in Australia, yes/no), education level (university degree vs no university degree), and work status (currently working, yes/no). Child characteristics included age and sex. Family characteristics included parents living together (yes/no) and siblings in the family (yes/no). Postcode was used to define living area (major cities, inner/outer regional or rural Australia) based on the Australian Bureau of Statistics Australian Statistical Geography Standard Remoteness Structure [35], and area-level socioeconomic status derived using the Australian Bureau of Statistics Socio-Economic Indexes for Areas (SEIFA) Index of Relative Socio-economic Advantage and Disadvantage (IRSAD) [36].

### Outcome measures

The outcomes for this study, assessed at baseline and the 6-month follow up (mid-intervention), have been described in more detail previously [28], all measures have been shown to have acceptable test–retest reliability [37–41], and are briefly described here.

#### Parental cognitions

*Parental knowledge* of child physical activity (8 items), screen time (6 items), and sleep (10 items) were assessed. Each individual behavior score was calculated as the sum of items answered correctly. A total score for parental knowledge of child movement behaviors was calculated as the sum of the scores for the individual behaviors (possible range 0–24, 24 = high level of knowledge) [37]. *Self-efficacy and confidence* [38], i.e., parent’s ability to engage their child in healthy movement behaviors and stick to rules and routines, for physical activity (mean of 2 items), sedentary behavior and screen time (mean of 5 items), sleep/bedtime routine (1 item) and rules and routines (1 item) were assessed. Each item had a possible range of 1–5, 5 = extremely confident. An overall score for parental self-efficacy and confidence was calculated as the mean of the scores for the individual behaviors. *Parenting confidence* was assessed with the Me-as-a-Parent tool and calculated as the sum of the 16 items (possible range 16–80, 80 = high regulation) [39]. *Ease of parenting*, i.e., how easy they are finding parenting their child at present, comprised of 1 item (possible range 1–5, 5 = very easy).

#### Parental behaviors

*Co-participation,* i.e., how often the parent engages in movement behaviors with their child, included co-participation in physical activity (1 item), sedentary behavior with focus on screen time (mean of 2 items), and sleep (1 item) (possible range 1–5, 5 = high co-participation) [38]. An overall co-participation score was calculated as the mean of the scores for the individual behaviors. *Parental modelling* of behaviors included habitual moderate- to vigorous-intensity physical activity (1 item, hours/week), screen time (1 item, hours/day), and sleep (1 item, hours/night) [40]. *Family rules* [38] included rules for physical activity (sum of 5 items, possible range 5–25), screen time (sum of 2 items, possible range 2–10), and sleep (1 item, possible range 1–5). A total score for family rules was calculated as the sum of the scores for the individual behaviors (possible range 8–40; 40 = more health-promoting rules). *Family routines* included routines for physical activity (1 item), screen time (1 item), and sleep (1 item) (categorical variables, yes/no), and a total score was calculated as the sum of the routines for the individual behaviors (possible range 0–3, 3 = having routines for all three behaviors). Presence of *screens in child’s bedroom* (including TV, electronic games, computer, phone, tablet) comprised of 1 item (categorical variable, yes/no).

#### Child developmental skills

*Motor skills* were assessed with the age-appropriate version of the Ages and Stages Questionnaire comprising 6 items (each scored as 10 = doing activity, 5 = sometimes, 0 = not yet). A summary score was calculated as the sum of the individual item scores (possible range 0–60, 60 = optimal score) [41]. *Emotional regulation,* e.g., how the child copes when things do not go their way, comprised of 4 items, and a summary score was calculated as the sum of the individual item (possible range 1–4, 4 = high regulation).

#### Participant engagement

For the intervention group only, objective engagement data over the 6-month period was collected using Web App Analytics and used to assess participant engagement with the *Let’s Grow* app. Specifically measures of total time spent in any part of the app (hours) and number of completed learning modules (out of a possible eight modules) were considered separately as measures of engagement.

### Statistical analyses

All statistical analyses were conducted in R version 4.0.3 (R Foundation for Statistical Computing), using complete case analysis and *P* values < 0.05 were considered statistically significant. Missing data ranged between 30–119 (2.6%−10.2%) for each of the intermediary outcomes. The effect of the intervention on each intermediary outcome was estimated using linear models (linear regression for continuous outcomes and logistic regression for binary outcomes). The models included group allocation, baseline value of the outcome to counteract imbalances at baseline, and the stratification factors (i.e., state and remoteness) to improve efficiency [42]. For each intermediary outcome we report the mean difference between the intervention and control groups estimated under the model alongside 95% confidence intervals (CI). We explored in the intervention group (*n* = 547) whether changes between baseline and follow up in numerical outcomes were associated with participant engagement with the *Let’s Grow* app. The cumulative score for the three binary variables representing family routines was used to assess change over time. The intermediary outcome of screens in the child’s bedroom was not included in this analysis given it is a binary variable. Engagement was defined as time spent in the app (hours) and number of completed modules.

The *Let's Grow* trial was powered for the primary outcome (compositional analysis based on accelerometry data on daily proportion of time spent in physical activity, sedentary behavior and sleep) [26]. We aimed to recruit 1100 families and assumed a 15% attrition rate at 6-months. Our target sample size (*n* = 467 families in each arm with complete data at 6 months) provides 90% power (two-tailed, alpha = 0.05) to detect a standardized effect (Cohen’s *d*) of 0.21 (low/medium effect size) in 6-month intermediary outcomes.

## Results

### Participant flow

**Fig. 1:**
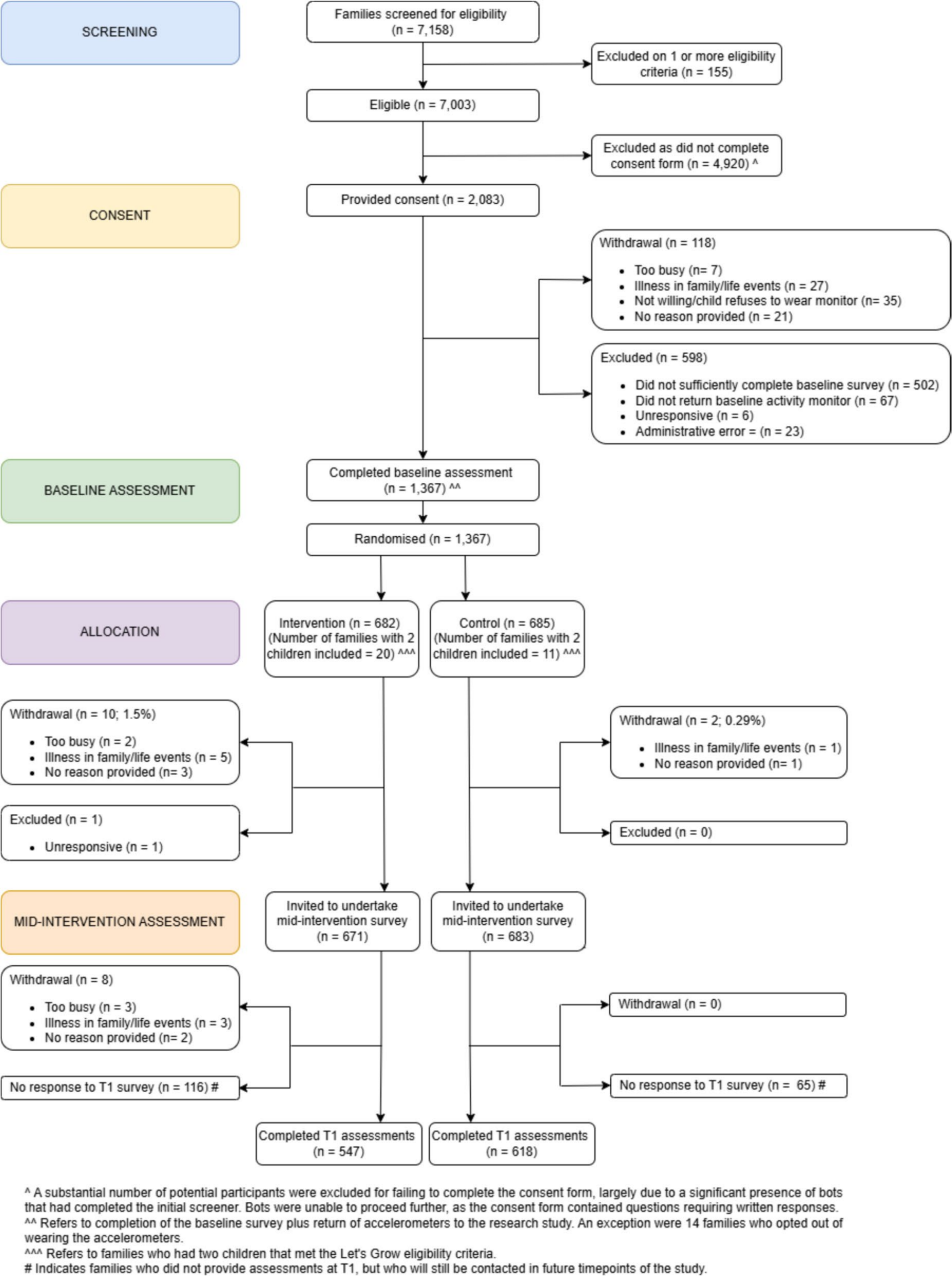
*Let’s Grow* trial CONSORT diagram until 6-month follow up (mid-intervention)

The flowchart of the *Let’s* Grow trial from baseline to the 6-month follow up is shown in Fig. 1. Initially 7158 families were screened for eligibility, out of which 7003 were deemed eligible. A substantial number of participants were excluded for failing to complete the consent form, likely due to a significant presence of bots that had completed the initial screener, resulting in 2083 families that provided written informed consent. In total, 1367 eligible parents completed the baseline measures (survey ± accelerometer) and were randomized (control, *n* = 685; intervention, *n* = 682). A total of 202 participants did not return the 6-month survey (135 intervention vs 67 control, *P* = 0.001), resulting in 85% (*n* = 1165; 618 control; 547 intervention) with data for at least one intermediary outcome at baseline and follow-up who were included in the analyses. Baseline characteristics were similar between those who returned and those who did not return the 6-month survey except for higher education level among the completers (73% vs 62% with a university degree, *P* = 0.006).

### Demographics and baseline characteristics

Baseline characteristics of the 1165 participants are presented in Table 1. The participating main carers were on average 34.8 (SD 4.4) years old (range: 21–55 years), the majority were mothers (99%), Australian born (78%), had English as their main language (94%), had a university degree (73%), were currently working (64%), lived together with a partner/spouse (93%), and lived in a major city (92%). The participating children were on average 27.1 (SD 4.1) months old, 53% were boys and 59% had siblings at home. There were no statistically significant differences in baseline characteristics between the groups at baseline (all *P* < 0.391).

**Table 1:** Baseline characteristics of the families in the *Let’s Grow* trial (*n* = 1165)^a^

	**All**	**Control** (*n* = 618)	**Intervention** (*n* = 547)
	n (%)	n (%)	n (%)
**Main parent**			
Age (years) (mean [SD])	34.8 [4.4]	34.7 [4.4]	34.9 [4.5]
Mother	1151 (98.8)	612 (99.0)	539 (98.5)
Australian born	901 (77.5)	480 (77.8)	421 (77.1)
English as main language	1087 (93.7)	575 (93.3)	512 (94.1)
University degree	849 (72.9)	448 (72.5)	401 (73.3)
Currently working	745 (64.0)	389 (62.9)	356 (65.2)
Living with partner/spouse	1084 (93.1)	578 (93.5)	506 (92.7)
Living in major cities	1068 (91.7)	568 (91.9)	500 (91.4)
Living in a high advantage area^b^	555 (47.8)	297 (48.2)	258 (47.3)
**Toddler**			
Age (months) (mean [SD])	27.1 [4.1]	27.2 [4.0]	27.0 [4.2]
Boys	612 (52.5)	333 (53.9)	279 (51.2)
Siblings at home	608 (59.1)	335 (61.0)	273 (56.9)

^a^Due to missing data, n varied slightly with number of missing data for individual variables ranging between 0 and 136. Data reported as n (%) unless otherwise stated

^b^Based on the area-level socioeconomic status derived using the Australian Bureau of Statistics Socio-Economic Indexes for Areas (SEIFA) Index of Relative Socio-economic Advantage and Disadvantage (IRSAD) [28]

### Intermediary outcomes at baseline

Table 2 presents the intermediary outcomes at baseline. Overall, parents reported *medium to high* scores for most parental cognitions and behaviors, as well as child developmental skills. Scores for the individual components of physical activity, screen time/sedentary behavior and sleep were similar for most constructs. An exception was parent knowledge where knowledge for physical activity (7.3 [SD 0.9] out of 8 for the intervention group and 7.3 [SD 0.9] for the control group) and sleep (7.5 [SD 1.8] out of 10 for the intervention group and 7.2 [SD 2.0] for the control group) were high, but knowledge for screen time (1.5 [SD 1.7] out of 6 for the intervention group and 1.4 [SD 1.7] for the control group) was low. There were no differences in the intermediary outcomes between the intervention and control groups at baseline, except for parental knowledge of child movement behaviors (15.8 [SD 2.9] vs 16.1 [SD 2.8], *P* = 0.012) and sleep (7.2 [SD 2.0] vs 7.5 [SD 1.8], *P* = 0.004) which were somewhat lower in the control group compared to the intervention group.

**Table 2:** Intervention effect on intermediary outcomes at 6-months (*n* = 1165)

		Control (*n* = 618)		Intervention (*n* = 547)					
	Possible range^a^	Baseline	Mid-intervention	Baseline	Mid-intervention	Intervention effect^b^	95% CI	*P*	*n*^c^
**Cognitions**									
*Knowledge*^d^	0–24	15.8 (2.9)	15.9 (2.8)	16.3 (2.8)	16.6 (2.8)	0.41	(0.15 to 0.67)	**0.002**	1065
Physical activity	0–8	7.3 (0.9)	7.2 (0.9)	7.3 (0.9)	7.4 (0.9)	0.12	(0.01 to 0.22)	**0.028**	1069
Screen time	0–6	1.4 (1.7)	1.2 (1.6)	1.5 (1.7)	1.3 (1.7)	0.03	(−0.13 to 0.18)	0.715	1070
Sleep	0–10	7.2 (2.0)	7.5 (1.9)	7.5 (1.8)	8.0 (1.9)	0.27	(0.10 to 0.45)	**0.003**	1072
*Self-efficacy and confidence*^e^	1–5	3.6 (0.7)	3.6 (0.7)	3.6 (0.7)	3.6 (0.7)	−0.02	(−0.08 to 0.05)	0.612	1066
Physical activity	1–5	3.6 (0.9)	3.6 (0.8)	3.6 (0.8)	3.5 (0.8)	−0.07	(−0.15 to 0.01)	0.088	1066
Sedentary behavior	1–5	3.5 (0.9)	3.4 (0.9)	3.6 (0.9)	3.5 (0.9)	0.02	(−0.06 to 0.10)	0.618	1066
Bedtime routine	1–5	4.1 (0.9)	4.1 (0.9)	4.1 (0.9)	4.1 (0.9)	−0.004	(−0.10 to 0.09)	0.928	1058
Rules and routines	1–5	3.8 (0.9)	3.8 (0.9)	3.8 (0.9)	3.8 (0.9)	−0.02	(−0.11 to 0.07)	0.655	1057
*General parenting*									
Parenting confidence	16–80	60.6 (7.7)	60.6 (7.8)	60.2 (7.5)	60.3 (8.4)	0.06	(−0.60 to 0.72)	0.858	1069
Ease of parenting	1–5	3.0 (0.8)	3.0 (0.9)	3.0 (0.8)	3.1 (0.8)	0.03	(−0.06 to 0.12)	0.476	1072
**Behaviors**									
*Co-participation*^f^	1–5	2.7 (0.6)	2.7 (0.6)	2.7 (0.6)	2.7 (0.6)	−0.02	(−0.07 to 0.03)	0.460	1061
Physical activity	1–5	3.4 (0.7)	3.3 (0.7)	3.4 (0.7)	3.3 (0.7)	−0.04	(−0.11 to 0.04)	0.330	1061
Sedentary behavior	1–5	2.2 (0.7)	2.6 (0.6)	2.2 (0.7)	2.2 (0.7)	0.02	(−0.05 to 0.08)	0.642	1060
Sleep	1–5	2.6 (1.5)	2.7 (1.4)	2.6 (1.4)	2.6 (1.4)	−0.05	(−0.16 to 0.06)	0.373	1058
*Parental modelling*									
MVPA (hours/week)	n/a	2.4 (2.5)	2.6 (3.9)	2.6 (3.2)	2.3 (2.5)	−0.36	(−0.73 to 0.02)	0.063	1057
Screen time (hours/day)	n/a	4.6 (2.8)	4.5 (2.9)	4.5 (2.6)	4.3 (2.7)	−0.11	(−0.42 to 0.20)	0.481	1057
Sleep (hours/night)	n/a	7.1 (1.1)	7.1 (1.2)	7.1 (1.2)	7.1 (1.2)	0.03	(−0.09 to 0.15)	0.640	1053
*Family rules*^g^	8–40	30.9 (3.9)	31.1 (3.9)	31.3 (3.8)	31.3 (3.8)	−0.04	(−0.39 to 0.31)	0.812	1062
Physical activity	5–25	18.7 (3.1)	18.8 (3.0)	18.9 (2.9)	18.8 (2.9)	−0.14	(−0.43 to 0.14)	0.321	1063
Screen time	2–10	8.4 (1.6)	8.4 (1.6)	8.5 (1.5)	8.5 (1.5)	0.05	(−0.10 to 0.20)	0.516	1046
Sleep	1–5	3.8 (1.1)	3.9 (1.0)	3.9 (1.0)	4.0 (0.9)	0.04	(−0.06 to 0.13)	0.470	1059
*Family routines*^h^	0–3	2.1 (0.8)	2.2 (0.8)	2.1 (0.9)	2.2 (0.8)	0.02	(−0.07 to 0.11)	0.640	1054
Physical activity (n, [%])	0–1	287 [46.7]	291 [50.3]	247 [45.2]	240 [49.8]	−0.09	(−0.35 to 0.18)	0.523	1058
Screen time (n, [%])	0–1	437 [71.2]	433 [74.8]	391 [71.5]	364 [75.4]	−0.05	(−0.34 to 0.24)	0.740	1059
Sleep (n, [%])	0–1	564 [91.3]	531 [90.3]	487 [89.0]	445 [90.3]	−0.26	(−0.70 to 0.18)	0.243	1082
*Environment*									
Screens in bedroom^i^ (n, [%])	0–1	*77* [12.7]	*93* [15.3]	*75* [13.9]	*77* [14.2]	−0.13	(−0.49 to 0.21)	0.451	1135
**Child developmental skills**									
Motor skills	0–60	49.8 (10.8)	52.1 (9.9)	49.4 (10.6)	52.5 (9.5)	0.73	(−0.25 to 1.71)	0.143	1064
Emotional regulation	1–4	2.6 (0.4)	2.6 (0.4)	2.6 (0.4)	2.6 (0.5)	0.02	(−0.03 to 0.06)	0.478	1074

Data presented as mean (SD) unless otherwise specified. SD, standard deviation; CI, confidence interval; MVPA, moderate- to vigorous-intensity physical activity

^a^Possible range for each item

^b^Coefficients associated to intervention effects estimated from linear regression for continuous outcomes and logistic regression for binary outcomes, adjusting for baseline levels of the outcome and the stratification factors

^c^Due to missing data, n varied slightly with number of missing data for individual variables ranging between 30 and 119

^d^Total score for parental knowledge regarding physical activity, screen time and sleep

^e^Mean overall score for parental confidence regarding physical activity, sedentary behavior, bedtime routine, and rules and routines

^f^Mean overall score for co-participation in physical activity, sedentary behavior, and sleep

^g^Total score for family rules regarding physical activity, screen time and sleep

^h^Total score for family routines regarding physical activity, screen time and sleep

^i^Indicator of presence of screens (TV, electronic games, computer, and phone or tablet) in the child’s bedroom

### Intervention effect on intermediary outcomes at 6-months

The intervention effect on the intermediary outcomes at six months is presented in Table 2. Parents in the intervention group had higher knowledge of child movement behaviors (mean difference = 0.41; 95%CI: 0.15 to 0.67; *P* = 0.002) compared to the control. The difference in total knowledge of movement behaviors was driven by shifts in two out of the three individual components, with higher scores for physical activity (mean difference = 0.12; 95%CI: 0.01 to 0.22; *P* = 0.028), and sleep knowledge (mean difference = 0.27; 95%CI: 0.10 to 0.45; *P* = 0.003). No significant differences were observed for any of the other parent cognitions and behaviors or child developmental skills.

### Association between participant engagement and change in outcomes from baseline

In terms of intervention uptake, 23 (4%) of the participants in the intervention group included in the analyses (*n* = 547) never logged in or used any of the app features but were included in the analyses based on intention to treat principles. Among those who logged in and used at least one of the app features (*n* = 524), over the six months the average time spent in the app was 34 min (SD 37; range 0–270 min), and the average number of completed modules was 2.8 (SD 3.1; range 0–8).

Table 3 presents the association between change in each intermediary outcome from baseline to 6-months and participant engagement defined as total time spent in the app and number of completed modules, respectively. Level of engagement was associated with several parental cognitions and behaviors but not with child developmental skills. Spending more time in the app was associated with higher scores for total knowledge of movement behaviors (β = 0.56; 95%CI 0.23 to 0.89; *P* = 0.001) and physical activity knowledge (β = 0.21; 95%CI: 0.07 to 0.36; *P* = 0.005), ease of parenting (β = 0.13; 95%CI: 0.02 to 0.24; *P* = 0.028), as well as total score for family rules (β = 0.51; 95%CI: 0.04 to 0.98; *P* = 0.035) and rules for screen time (β = 0.22; 95%CI: 0.02 to 0.43; *P* = 0.031). Similarly, completing more modules was also associated with *higher* scores for parental knowledge of movement behaviors (β = 0.09; 95%CI: 0.02 to 0.15; *P* = 0.008) and physical activity (β = 0.03; 95%CI: 0.00 to 0.06; *P* = 0.027), confidence (β = 0.18; 95%CI: 0.02 to 0.35; *P* = 0.026), and total score for family rules (β = 0.11; 95%CI: 0.02 to 0.20; *P* = 0.019), as well as *less* parental screen time (β = −0.08 h/day; 95%CI: −0.16 to −0.00; *P* = 0.04, equivalent to 5 min less per day).

**Table 3:** Association between participant engagement and change in intermediary outcomes from baseline (intervention group, *n* = 547)^a^

Intermediary outcomes	**Association between change in intermediary outcomes and engagement**^**b**^					
	**Time spent in the app over the 6-months (hours)**			**Completed modules** **over the 6-months**		
	Beta	(95% CI)	*P*	Beta	(95% CI)	*P*
**Cognitions**						
*Knowledge*^c^	0.56	(0.23 to 0.89)	**0.001**	0.09	(0.02 to 0.15)	**0.008**
Physical activity	0.21	(0.07 to 0.36)	**0.005**	0.03	(0.00 to 0.06)	**0.027**
Screen time	0.17	(−0.03 to 0.39)	0.101	0.03	(−0.01 to 0.07)	0.167
Sleep	0.18	(−0.06 to 0.41)	0.135	0.03	(−0.02 to 0.07)	0.236
*Self-efficacy & confidence*^d^	−0.02	(−0.10 to 0.06)	0.652	0.00	(−0.01 to 0.02)	0.705
Physical activity	−0.02	(−0.13 to 0.09)	0.711	0.01	(−0.02 to 0.03)	0.523
Sedentary behavior	−0.06	(−0.16 to 0.05)	0.319	−0.01	(−0.03 to 0.02)	0.634
Bedtime routine	0.04	(−0.08 to 0.16)	0.493	0.01	(−0.01 to 0.03)	0.401
Rules and routines	0.09	(−0.03 to 0.22)	0.153	0.02	(−0.01 to 0.04)	0.129
*General parenting*						
Parenting confidence	0.76	(−0.06 to 1.59)	0.071	0.18	(0.02 to 0.35)	**0.026**
Ease of parenting	0.13	(0.01 to 0.24)	**0.028**	0.01	(−0.01 to 0.03)	0.289
**Behaviors**						
*Co-participation*^e^	−0.02	(−0.09 to 0.06)	0.659	−0.00	(−0.02 to 0.01)	0.584
Physical activity	0.06	(−0.04 to 0.16)	0.268	0.01	(−0.01 to 0.03)	0.399
Sedentary behavior	0.00	(−0.09 to 0.09)	0.986	−0.00	(−0.02 to 0.02)	0.882
Sleep	−0.11	(−0.25 to 0.04)	0.162	−0.02	(−0.05 to 0.01)	0.136
*Parental modelling*						
MVPA (hours/week)	−0.11	(−0.52 to 0.30)	0.595	0.01	(−0.07 to 0.09)	0.826
Screen time (hours/day)	−0.30	(−0.69 to 0.09)	0.132	−0.08	(−0.16 to −0.00)	**0.039**
Sleep (hours/night)	0.05	(−0.11 to 0.21)	0.554	0.01	(−0.03 to 0.04)	0.690
*Family rules*^f^	0.51	(0.04 to 0.98)	**0.035**	0.11	(0.02 to 0.20)	**0.019**
Physical activity	0.21	(−0.17 to 0.60)	0.282	0.07	(−0.01 to 0.14)	0.090
Screen time	0.22	(0.02 to 0.43)	**0.031**	0.03	(−0.01 to 0.07)	0.147
Sleep	0.07	(−0.07 to 0.20)	0.348	0.02	(−0.01 to 0.05)	0.214
*Family routines*^g^	0.00	(−0.12 to 0.12)	0.980	0.001	(−0.02 to 0.03)	0.526
**Child developmental skills**						
Motor skills score	−0.19	(−1.62 to 1.23)	0.791	−0.00	(−0.29 to 0.28)	0.973
Emotional regulation	0.01	(−0.05 to 0.07)	0.742	−0.00	(−0.01 to 0.01)	0.915

^a^Due to missing data, n varied slightly with number of missing data for individual variables ranging between 33 and 82. The categorical outcome screens in the child’s bedroom was not included in these analyses. SD, standard deviation; CI, confidence interval; MVPA, moderate- to vigorous-intensity physical activity

^b^The regression models included change in the intermediary outcome (calculated as outcome at follow up minus outcome at baseline) regressed on level of user engagement defined as total time spent in the app (hours during the 6-month period) and number of completed modules (i.e., behavior change component of the app)

^c^Total score for parental knowledge regarding physical activity, screen time and sleep

^d^Mean overall score for parental confidence regarding physical activity, sedentary behavior, bedtime routine, and rules and routines

^e^Mean overall score for co-participation in physical activity, sedentary behavior, and sleep

^f^Total score for family rules regarding physical activity, screen time and sleep

^g^Total score for family routines regarding physical activity, screen time and sleep

## Discussion

This study aimed to investigate potential differences in the intermediary outcomes for behavior change between the intervention and control group in the *Let’s Grow* trial after 6-months. Overall, parents reported *medium to high* scores for knowledge, confidence, and rule setting for all movement behaviors at baseline, indicating a possible ceiling effect of these measures. Findings showed that at the mid-intervention phase, the *Let’s Grow* intervention appeared to have a small effect on parental knowledge around physical activity and sleep, but parents might be in more need of support related to their knowledge of screen time recommendations and impacts for children or may require more time to complete and consolidate knowledge from the current intervention. There was no evidence of effect on any of the other intermediary outcomes. The results also indicate that level of engagement with the intervention impacted the effectiveness of the intervention on some intermediary outcomes.

Even though parents reported high levels of knowledge prior to the commencement of the trial, our findings indicate that the *Let’s Grow* intervention can further positively influence parental knowledge of movement behaviors. However, mid-way through the intervention, the observed effects were rather small (~ 1% increase), and parents might need more time or other types of support to improve parent behaviors and child developmental skills. Moreover, the effect on parental knowledge of movement behaviors was driven by an overall positive shift in knowledge of physical activity and sleep, but not screen time. This is interesting considering that parents reported high scores for physical activity and sleep knowledge at baseline but low scores for screen time, which theoretically would represent more opportunity for change. Parents’ knowledge of the guidelines is important as it has been shown to be associated with greater compliance with both individual and combined movement guidelines for toddlers [29], and increasing parental knowledge around screen time has been shown to have implications for child behaviors. To illustrate, data from the INFANT trial identified maternal knowledge as the primary mechanism explaining the significant intervention effect on children’s reduction of television viewing at 19 months of age [43]. This also explained the maintained intervention effect on television viewing post intervention [44], as better maternal knowledge was associated with less television viewing time in their toddlers which persisted into the preschool years. The null effect on screen time knowledge in our study could reflect the aspects of the intervention which parents engaged with (or did not engage with). Considering that the intervention focused on the interplay between the different movement behaviors this is however difficult to distinguish. Nevertheless, previous studies have shown that contemporary parents view screen time as a part of daily life while also struggling to find a screen time balance for their children [45]. This suggest there is much scope to work on increasing parent knowledge around screen time for children, and it is possible that parents might need support specifically targeting screen time.

While there is a paucity of studies reporting on intermediary intervention outcomes, previous studies that have investigated the effect of childhood health behavior interventions on parental practices and cognitions have shown mixed results. For instance, similarly to our results, Hammersley et al. [46] reported no effect on parental self-efficacy nor modeling for screen time, physical activity or sleep for parents to 3–5-year-old children following an obesity prevention intervention. In contrast, positive intervention effects on parenting practices such as screen time parenting practices [22, 23], physical activity role modeling [23, 24], and lower use of social co-viewing practices [22] have been shown in studies targeting preschool- and school-aged children. The discrepancies in results could potentially be explained by differences in child age (ranging from 2 to 12 years of age) or intervention characteristics, as previous interventions have been delivered face-to-face, and have aimed to improve both movement behaviors and nutrition [22, 23] or only physical activity [24] or sleep [25], while *Let’s Grow* was delivered remotely via an app and focused on all movement behaviors.

Another potential explanation for the modest results observed in our study is the level of engagement with the intervention. The link between engagement in interventions targeting health behaviors and intervention effectiveness has previously been established in other populations (e.g., [47–50]). However, the reporting of engagement in digital interventions targeting parents of young children is largely lacking, thus there is little guidance on expected or desired levels of engagement. To the best of our knowledge, this is the first study to examine associations of engagement with the effect of a digital intervention to promote young children’s movement behaviors on the intermediary outcomes. In line with studies on outcomes in other populations (e.g., [47–50]), our findings also showed that higher engagement was associated with more favorable outcomes. Specifically, higher engagement was associated with small improvements in several intermediary outcomes including parental knowledge of movement behaviors and physical activity, confidence and ease of parenting, total scores for family rules as well as rules specific for screen time. Finally, higher engagement was also associated with improved modeling of behavior with parents having less screen time (5 min less per day), despite the additional screen time inherent in an intervention delivered via an app. Altogether, this shows that engagement with a digital movement behavior intervention could have a positive impact on screen related practices in the home environment and confirms findings from other studies showing that engagement is important for intervention impact [47–50].

From an intervention implementation perspective, it is also relevant to discuss the overall level of engagement; however, to date, there is no universal criteria for ‘good’ or ‘required’ engagement for intervention efficacy and the vast majority of mHealth interventions fail to report on engagement leaving us to rely largely on industry app data. Initial engagement in our study (96% logged in and used at least one app feature) can be considered very high in comparison to industry reports app usage as 25% of all downloaded apps are engaged with just once in the first six months [51]. It is similar to findings from another study in the early childhood population, which reported comparable measures of engagement in a digital health intervention [52]. Interestingly, the high initial engagement does not seem to reflect parental engagement over time, as the average number of modules completed half-way through the intervention was just under three out of the eight modules, falling short of what might be expected at that point in the intervention. Retaining participants’ engagement in digital health interventions has been reported to be challenging [53, 54] and previous studies have shown a decline in usage over time (e.g., [55–57]), as do industry reports of patterns of app usage [51]. Although the digital format offers a convenient way to provide timely information to parents while also enabling participation regardless of geographic location, engagement issues highlight potential challenges for this mode of delivering support to parents. Moreover, a recent review on parent engagement in child-focused interventions in healthcare described parent engagement as a relational process, where building and maintaining relationships within the intervention setting are important to foster parent engagement [58], something that is challenging when the intervention is delivered anonymously from a distance via an app. These considerations should be taken into account when weighing up the benefits of reach of digital interventions with the potential limitations of reduced engagement.

### Strengths and limitations

This is one of few studies to examine intervention effects on the targeted intermediary outcomes (i.e., parental cognitions and behaviors, child developmental skills) to improve movement behaviors in young children. The study has several strengths including the randomized controlled design, the large sample size, and comprehensive battery of parental behavior and cognition intermediary outcomes investigated, as well as objective data on participant engagement with the intervention. The study also has some limitations. First, we utilized mid-intervention data, thus our results do not capture the full length of the intervention; however, similar interventions have been considerably shorter (e.g., [46]). Our study provides important information for determining whether the intervention is meeting objectives and can help identify areas in which the intervention might need to be enhanced. Second, parent and child intermediary outcomes were self- (or proxy-) reported and thus results may be subject to reporting biases (i.e., recall bias and social desirability), and certain instruments do not have validity evidence; however, all have been shown to have acceptable test–retest reliability [37–41]. As stated above, we report intervention effects on a comprehensive battery of outcomes, which have been identified as important intermediary outcomes in early childhood interventions [14]; however, testing multiple outcomes might have increased the likelihood of chance findings. Finally, the limited intervention effect may be attributed to the high education level of participants and increased retention of higher educated parents, that could have influenced their baseline knowledge and behaviors, reducing the opportunity for improvement from the intervention. We did however run a sensitivity analysis adjusting for education level in the main model, and results remained the same. The high education level of participants, and the majority of which were mothers also limits the generalizability of our findings to other population groups.

### Societal relevance

We report the effects of a comprehensive parenting intervention program aiming to improve all movement behaviors in toddlers on the intermediary outcomes that theoretically lead to intervention effects. Our findings suggest minimal impact on these intermediary outcomes at the mid-intervention point, which indicates that parents might need more support to engage with the intervention or more time for change to be affected. Moreover, the low intervention impact could potentially be explained by a ceiling effect on several measures already at baseline. Our findings showed that engagement appeared to predict change in several of the outcomes, suggesting the importance of engagement [59] and highlights the need to focus on engaging parents as a core part of intervention delivery. Another important aspect is the specific intervention content to which participants are exposed (e.g., whether participants were exposed to topics covering all targeted behaviors) and how this exposure impacts the results. Given the vast majority of participants visited app pages exposing them to content on all three targeted behaviors, we were unable to analyze results stratified by specific behavior message exposure, as is often the case in multi-behavioral interventions. However, this remains an important topic for future research. Moreover, the intervention was conducted during the COVID pandemic, which likely added additional pressures on families and might have limited their ability to engage with the program, and consequently also the effect of the intervention. This makes it difficult to accurately predict how well this intervention would engage parents in a more typical era.

It is yet to be determined whether the observed effects on the intermediary outcomes of parental knowledge after 6-months are sufficient to achieve improvements in child movement behaviors, how intervention engagement will impact the primary outcomes of the trial, and whether the intervention is cost-effective. These questions will be tested when reporting the effect of the main outcome of the study following trial conclusion, as described in the study protocol [28]. Altogether, these results provide an increased understanding of the mechanisms of behavior change, which is important for future development of more impactful interventions.

## Conclusions

Our findings indicate that the *Let’s Grow* intervention can positively influence parental knowledge of child movement behaviors in the first 6-months of use, though changes were small, and parents might be in need of more time and support to improve cognitions and behaviors related to their child’s sedentary behavior, particularly screen time, as well as child developmental skills. Engagement with the intervention appears to be important to enhance intervention effects, underscoring the importance of strategies to optimize engagement. Future studies should investigate how to strengthen parental self-efficacy and confidence in general and specific to movement behaviors and child developmental skills, as well as strategies to optimize engagement.

## Data Availability

The datasets used and/or analyzed during the current study are available from the corresponding author upon reasonable request.

## Abbreviations

MVPA: Moderate- to vigorous-intensity physical activity
CI: Confidence Intervals
SD: Standard Deviation
SEIFA: Socio-Economic Indexes for Areas
